# Evaluation of impact of measles rubella campaign on vaccination coverage and routine immunization services in Bangladesh

**DOI:** 10.1186/s12879-016-1758-x

**Published:** 2016-08-12

**Authors:** Md Jasim Uddin, Gourab Adhikary, Md Wazed Ali, Shahabuddin Ahmed, Md Shamsuzzaman, Chris Odell, Lauren Hashiguchi, Stephen S. Lim, Nurul Alam

**Affiliations:** 1 Centre for Equity and Health Systems, icddr,b, Mohakhali, Dhaka 1212 Bangladesh; 2 Institute for Health Matrices and Evaluation (IHME), University of Washington, Seattle, USA; 3 Centre for Population, Urbanization and Climate Change, icddr,b, Mohakhali, Dhaka 1212 Bangladesh

**Keywords:** Campaign, Coverage, Measles, Rubella, Vaccination, Bangladesh

## Abstract

**Background:**

Like other countries in Asia, measles-rubella (MR) vaccine coverage in Bangladesh is suboptimal whereas 90–95 % coverage is needed for elimination of these diseases. The Ministry of Health and Family Welfare (MOHFW) of the Government of Bangladesh implemented MR campaign in January-February 2014 to increase MR vaccination coverage. Strategically, the MOHFW used both routine immunization centres and educational institutions for providing vaccine to the children aged 9 months to <15 years. The evaluation was carried out to assess the impact of the campaign on MR vaccination and routine immunization services.

**Methods:**

Both quantitative and qualitative evaluations were done before and after implementation of the campaign. Quantitative data were presented with mean (standard deviation, SD) for continuous variables and with proportion for categorical variables. The overall and age- and sex-specific coverage rates were calculated for each region and then combined. Categorical variables were compared by chi-square statistics. Multiple logistic regression analysis were performed to estimate odds ratios (OR) and 95 % confidence intervals (CI) of coverage associated with covariates, with adjustment for other covariates. Qualitative data were analyzed using content analysis.

**Results:**

The evaluations found MR coverage was very low (<13 %) before the campaign and it rose to 90 % after the campaign. The pre-post campaign difference in MR coverage in each stratum was highly significant (*p* < 0.001). The campaign achieved high coverage despite relatively low level (23 %) of interpersonal communication with caregivers through registration process. Child registration was associated with higher MR coverage (OR 2.91, 95 % CI 1.91–4.44). Children who attended school were more likely to be vaccinated (OR 8.97, 95 % CI 6.17–13.04) compared to those who did not attend school. Children of caregivers with primary or secondary or higher education had higher coverage compared to children of caregivers with no formal education. Most caregivers mentioned contribution of the campaign in vaccination for the children not previously vaccinated.

**Conclusions:**

The results of the evaluation indicated that the campaign was successful in terms of improving MR coverage and routine immunization services. The evaluation provided an important guideline for future evaluation of similar efforts in Bangladesh and elsewhere.

## Background

Measles and rubella are among the most infectious diseases of humans. High level of herd immunity is required for its elimination [1]. Seroprevalence studies suggest that coverage in the range of 90–95 % is needed [2]. Measles and rubella are vaccine-preventable diseases with similar symptoms and are frequently confused with each other. Both viruses cause rash and fever [3]. Measles can be deadly for children with poor nutrition and weakened immune systems. Rubella is also very contagious but causes relatively mild disease in children; in pregnant women, rubella can lead to miscarriage or severe birth defects (congenital rubella syndrome), including blindness, deafness, and heart problems [4]. Rubella and measles are a public health problem in poor countries in Africa and Asia, including Bangladesh, where uptake of the measles and rubella vaccine is relatively low and increasing access to immunization through large-scale vaccination campaigns can significantly reduce deaths and illnesses [5].

By the end of 2009, all WHO regions achieved either measles-rubella control or elimination goals [3]. Pan American Health Organization (PAHO) and European Regional Office (EURO) instituted combined measles and rubella elimination goals. In the PAHO region, it is believed that this elimination goal has been achieved, and a certification committee has been formed. In regions under Western Pacific Regional Office (WPRO), the goal was to eliminate measles by 2012. African Regional Office (AFRO) adopted a pre-elimination goal in 2008, which was very close to elimination. South East Asian Regional Office (SEARO) has recently transitioned from a mortality reduction goal to an elimination goal.

Like other countries in Asia, measles-rubella (MR) vaccine coverage in Bangladesh is suboptimal [6]. In 2011, the routine surveillance system detected serological and expanded programme on immunization (EPI)-linked rubella incidence in Bangladesh among 37 per million individuals. The previous year--2010--had an epidemic and a rubella incidence rate of 87 per million individuals. The objective of vaccination of <15 children is to elimination of MR. Around 80 % of the measles and rubella cases were less than 15 years of age in 2011 [7]. The finding suggests that national-level MR campaign covering children with aged 9 months to <15 years may substantially increase the coverage and reduce both rubella and measles virus circulation and enable to achieve measles and rubella elimination goal.

Measles vaccination campaigns were undertaken in Bangladesh in 2005, 2006, and 2010 to boost measles vaccination coverage. According to the reports, the routine measles coverage increased from below 60 % to above 80 % after the 2005–2006 measles campaign. The post- campaign survey conducted in 2010, reported that the campaign had improved measles coverage [8]. The impact evaluations of those campaigns were not very systematic in terms of assessment of workload, effects of the campaign on activities of routine EPI and other health systems. The target population of previous measles campaigns was very narrow-children aged 9–12 months compared to the target population of the MR campaign in 2014 among children aged 9 months to <15 years.

The Gavi Alliance is investing more than US$ 600 million in the fight against measles and rubella through large-scale campaigns [9]. The Alliance targets over 700 million children in 49 countries aged 9 months to 14 years to immunize against measles-rubella [10]. The Gavi Alliance supported MR campaign in Bangladesh in 2014 to supplement the introduction of routine MR vaccination [11]. The Ministry of Health and Family Welfare (MOHFW) of the Government of Bangladesh (GoB) implemented the MR campaign nationally in January-February 2014 targeting more than 52 million children aged 9 months to <15 years [12] to increase MR vaccine coverage. Schools and other educational institutions and routine EPI centres were used for sessions on MR vaccination. Students remained absent at school during campaign were vaccinated at routine immunization outreach centers. The campaign was conducted nationally and, to date, is the largest MR campaign conducted globally. To guide continued measles and rubella elimination activities, an effective evaluation of the 2014 MR campaign was critical. This article highlighted the impact of the MR campaign on improving MR vaccine coverage and on the routine EPI in Bangladesh.

## Methods

Mixed method approaches were employed at various levels of the health system of Bangladesh. Assessments were conducted in 2013 before the MR campaign and in 2014 after the campaign.

### Study areas

The evaluation focused on selected locations stratified by urban/rural and low/high performing areas. The post-campaign survey was nationally representative. Bangladesh is divided into seven administrative divisions (regions). These divisions consist of 64 districts, which are further subdivided into 485 upazillas (sub-districts). We had purposively selected two divisions, one high performing (namely Rajshahi) and one low performing (namely Sylhet), from the seven divisions for pre campaign evaluation. Rajshahi is in the western part, and Sylhet in the northeastern part of Bangladesh. From the two divisions we selected one rural district and one urban city corporation each. In Rajshahi Division, the rural district is Joypurhat and the urban city corporation is Rajshahi City Corporation. According to the annual coverage evaluation survey (CES) 2011, Joypurhat district and Rajshahi City Corporation have the highest EPI coverage within their division. In the Sylhet division, Sylhet district and Sylhet City Corporation have the lowest EPI coverage within their division. Thus, we selected the highest performing Joypurhat district and Rajshahi city, and the lowest performing Sylhet district and Sylhet City.

### Sampling

We conducted separate coverage surveys both pre and post campaign. Pre-and-post-campaign surveys were necessary to understand how population-level vaccine coverage had changed as a result of the campaign.

To minimize costs of conducting the survey work for the pre-campaign survey we focused on four geographies divided between urban/rural and high performing/low performing areas. As stated, the post campaign survey was nationally representative so that population-level vaccine coverage could be estimated. We purposefully sampled the same geographies included in the pre-campaign survey to allow measurement of change in vaccine coverage as a result of the campaign.

#### Sampling frame for pre-campaign survey

Since selection of locations were intentional based on administrative results, population denominator was adjusted applying the appropriate sampling rate. Each sub-district is divided into unions and each union into 24 EPI clusters, each of around 200 households (or 1000 population). The list of EPI clusters is quite complete and up-to-date, and is available at the sub-district EPI offices. This list served as the sampling frame for the first stage of selection of EPI clusters in each stratum.

In total 1,735 households were sampled in the pre-campaign survey. A stratified two-stage random cluster sampling design was followed to minimize travel costs and time and to ease supervision. The first stage was the random selection of EPI clusters, and the second stage was selection of households with eligible children. The Coverage Evaluation Survey (CES) commissioned by MOHFW to a third party every year provides estimates of EPI coverage by antigen at division and district levels, and cities separately. The CES estimates were used to identify two rural districts—namely Joypurhat and Sylhet (excluding the city), and two cities—Rajshahi and Sylhet with the highest and the lowest EPI coverage in the respective divisions. The minimum required sample size was estimated for each stratum in order to obtain statistical significance of difference in MR coverage 10 % points or more between the pre- and post-campaign surveys at *p* = 5 % with 80 % power of the test.

#### Sampling framework for post campaign survey

The post campaign survey was designed to nationally representative, and to provide sub-national (administrative division) estimates. Our post-campaign survey estimates allowed us to compare with the pre-campaign survey estimates to assess the change in measles and rubella vaccine coverage due to the MR campaign. The total sample size of the post-campaign survey was 4,510.

## Data collection

Following techniques were used for data collection for the evaluation:

### Pre and post-campaign surveys

Before implementation of the MR campaign, a survey was conducted with the primary caregiver of children aged 9 months to < 15 years. Also after the MR campaign was completed, we conducted a post-campaign survey, using the same procedure as pre-campaign survey. Data included: status of MR vaccination among children, key demographic and household socio-economic status (SES) data, demand-side constraints and perceptions about the campaign.

### Qualitative technique

#### Key informant interviews

A total of 58 purposively selected key informant interviews (KIIs) were conducted after the MR campaign, following coverage surveys at the national, district, and facility levels. Issues explored through KII include: human resource and workload, demand generation, and supply of vaccine and other related commodities supply. We sampled key informants from national, district and sub-district levels.

#### Data analysis

In the post-campaign survey, probability of selection of the EPI centres at division level and the probability of successful interview at cluster level within divisions varied, as such sampling weights (reciprocal of the selection probability) were estimated to compute weighted coverage rates for each division and all divisions combined. Data were presented with mean (standard deviation, SD) for continuous variables and with proportion for categorical variables. The overall and age- and sex-specific coverage rates were calculated for each division and then combined. Categorical variables were compared by chi-square statistics. Multiple logistic regression analyses were performed to estimate odds ratios (OR) and 95 % confidence intervals (CI) of coverage associated with covariates, with adjustment for other covariates. Qualitative data were analyzed using content analysis.

## Results

### Socio-demographic characteristics of respondents

Respondents in the pre- and post campaign surveys were mostly mothers/female caregivers of children (>98 % in rural and in urban areas) with a few exceptions. Majority of them were aged 25–44 years in each survey. The mean age of the respondents from the high-performing division was little lower than that from low-performing division. The difference in secondary or more education between the high and the low performing divisions was larger than the difference between the urban and rural strata within the divisions.

### Impact of MR campaign on MR vaccination coverage

**Fig. 1:**
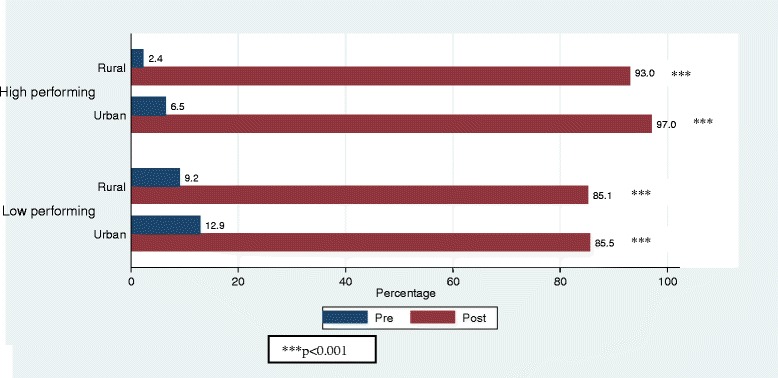
MR coverage in pre- and post campaign surveys in high and low performing divisions

As expected MR coverage, defined as recall of having received MR vaccination before and during the campaign, was very low in each stratum in the pre-campaign survey, followed by a sharp rise in the post-campaign survey (Fig. 1). The pre-post difference in MR coverage in each stratum was highly significant (*p* < 0.001).

### MR coverage by division and age group of children

**Fig. 2:**
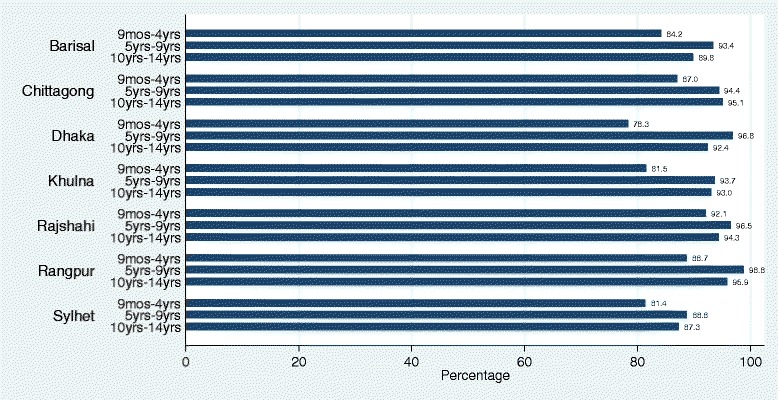
MR coverage by administrative division and by age group of children

National MR Coverage in the post-campaign survey was 90 % (95 % CI: .89–.92). Although overall coverage was high, there were some important differences to highlight by geography and age group of children. Coverage varied by division, with Rajshahi having 94 % (95 % CI: .93–.96) coverage while Sylhet had only 83 % (95 % CI: .83–.88). The coverage of other divisions were 89 % (95 % CI: .86–.92) in Barisal, 91 % (95 % CI: .89–.95) in Chittagong, 89 % (95 % CI: .87–.93) in Dhaka, 90 % (95 % CI: .86–.93) in Khulna and 94 % (95 % CI: .93–.97) in Rangpur. The findings revealed that MR coverage was higher among school going age group (5–14 years) children compared to non-school going group of children (Fig. 2).

### MR coverage by potential correlates

There were some small, but significant differences in the MR coverage by child’s and household’s characteristics (Table 1). The campaign achieved high coverage despite relatively low level (23 %) of personal communication with caregivers through the registration process. Child registration was associated with higher MR coverage (OR 3.31, 95 % CI 2.37–4.63). Child’s age and sex were associated with MR coverage. Children who attended school were more likely to be vaccinated (OR 8.97, 95 % CI: 6.17–13.04), compared to those who did not attend school. Children of caregivers with primary or secondary or higher education had higher coverage compared to children of caregivers with no formal education. Though household asset quintile and type of residence were not associated with coverage but living in an administrative division was associated with high or low coverage.

**Table 1:** Levels and differentials in MR coverage and odds ratio^a, b^ (OR) of MR vaccination for selected covariates in post-campaign survey

Name of the covariate	Number of children	Coverage rate (%)	Logistic regression model estimates		
			Adjusted OR^a, b^	95 % CI	*p*-value
Child’s registration prior to campaign					
Not registered	3401	87.1	1.00	REF	
Registered	1096	95.2	3.31	(2.37–4.63)	<0.001
Sex of the child					
Male	2415	88.0	1.00	REF	
Female	2081	90.3	1.21	(0.99–1.48)	0.062
Age of the child					
9 months – 4 years	1184	82.3	1.00	REF	
5–9 years	1637	92.8	1.91	(1.26–2.89)	0.002
10–14 years	1673	90.0	1.28	(0.78–2.11)	0.324
Child’s school attendance					
Did not attended	185	57.8	1.00	REF	
Attended	2821	93.7	8.97	(6.17–13.04)	<0.001
Minor (aged ≤5 years)	1284	84.1	3.84	(2.28–6.48)	<0.001
Mother’s education level					
None	1193	84.8	1.00	REF	
Primary (up to V)	1581	89.7	1.44	(1.10–1.89)	0.008
Secondary and higher	1723	91.4	1.63	(1.21–2.20)	0.001
Household asset quintile					
Lowest	914	87.4	1.00	REF	
Second	886	89.6	1.21	(0.88–1.65)	0.233
Middle	901	89.9	1.20	0.85–1.68)	0.300
Fourth	950	90.2	1.23	(0.85–1.80)	0.263
Highest	844	88.0	1.11	(0.84–2.52)	0.184
Administrative division					
Barisal	451	89.6	1.00	REF	
Chittagong	428	91.4	1.45	(0.84–2.52)	0.184
Dhaka	414	89.4	1.04	(0.65–1.68)	0.862
Khulna	378	89.7	1.09	(0.65–1.82)	0.738
Rajshahi	986	94.2	1.92	(1.18–3.14)	0.009
Rangpur	474	93.9	2.04	(1.24–3.36)	0.005
Sylhet	1366	82.4	0.59	(0.39–0.91)	0.016
Type of residence					
Rural	3570	89.6	1.00	REF	
Urban	927	87.0	1.02	0.68–1.53	0.908

^a^The dependent variable was coded ‘1’ if child was vaccinated, ‘0’ otherwise

^b^OR was adjusted for clustering of vaccination of children of the same clusters

### Reasons for not vaccinating during MR campaign

In our post-campaign survey, 17 % of primary caregivers of unvaccinated children did not know about the campaign with some geographic variations (Table 2). This was higher in Sylhet and Chittagong, where vaccination coverage was consistent low, than Rangpur, Dhaka and Khulnar. Around one third of caregivers of unvaccinated children cited child’s sickness at the time of vaccination and another one-third cited fear of side effects as reasons for non-vaccination.

**Table 2:** Percentage distribution of reasons for not vaccinating children under the MR campaign, by administrative divisions

Reasons for not vaccinating^a^	Total	Barisal	Chittagong	Dhaka	Khulna	Rajshahi	Rangpur	Sylhet
Unaware of campaign	17.11	4.26	19.44	9.52	7.69	7.02	17.86	24.58
Fear of side effects	31.96	40.43	27.78	30.95	35.9	42.11	21.43	29.24
Child was sick	30.52	40.43	38.89	59.52	25.64	17.54	28.57	26.27
Others	22.68	17.02	19.44	0	30.77	36.84	32.14	22.46
n	485	47	36	42	39	57	28	236

^a^Multiple responses

### Impact of MR campaign on routine EPI

#### Coverage of measles containing vaccine (MCV) before and after the campaign

Routine coverage of MCV was increased after the campaign. The increase was the highest from 80 % in the pre-campaign to 95 % in the post-campaign in rural stratum of Rajshahi division. On contrary, the increase was lowest 2 % points in the Rajshahi city. In Sylhet division, the increases in rural and urban strata were 3 % and 4 % respectively (data not shown). The results from qualitative component showed that the MR campaign had positive impact on routine EPI services in Bangladesh. Followings are the details about this:

##### Increased public awareness and acceptance

Findings showed that during preparation for MR Campaign, the GoB was concerned that persistent fears in the population about the adverse events and child death from a Vitamin A provided in the campaign in March 2013 would impact on demand for MR vaccine. Our findings suggest, however, that the MR campaign helped to reshape perceptions. One rural service provider stated:

*Our routine EPI coverage was low after the rumor of Vitamin A plus campaign. The situation changed in the community after the MR campaign. Specifically, when people came to the MR campaign sessions, they gained a better understanding and reduced their misconceptions. They realized that these vaccines have been introduced for their welfare. We also mentioned to the guardians that Vitamin A plus campaign’s adverse news which were broadcasted were nothing but rumors*.

##### Improved provider-caregiver communication

This campaign improved the communication between service providers and caregivers during the preparation phase of the campaign as a result of the door-to-door registration process. This door-to-door registration process increased provider-caregiver communication, and had the potential to improve communication for routine EPI.

##### Improved logistics

During the MR campaign, many logistics (e.g., vaccine carriers, ice packs, ice lining refrigerators, deep freezers, and vehicles) were either repaired or newly purchased. Although most of these logistics were very costly, they were critical for preserving the quality of the vaccines. These logistics would also be available to the routine EPI program.

##### Strengthened inter-sectoral coordination

In order to conduct such a large nationwide program, proper coordination and integration at different levels and across sectors was required. These lessons learned and experience can be incorporated in the EPI sector for future immunization campaigns and large scale health interventions.

##### Demand on health worker time and implications on routine activities

The MR campaign was designed to minimize interference to routine EPI activities by scheduling campaign activities for 5 days of the week with routine EPI activities for 2 days of the week. It is important to note that this resulted in a significant increase in workload for EPI service providers as evidence by the EPI provider survey conducted. However, findings from key informant interviews suggest that health workers were highly motivated to work extra hours.

##### Catch up immunizations for MR vaccine recipients

Mass immunization campaigns offer an important opportunity to catch up or provide booster doses for other antigens for children receiving the campaign vaccine [12]. The MR campaign was not explicitly designed to catch up on other antigens. Findings indicated that some health workers did use the opportunity for catch up. Among children aged 9–35 months, 1.3 % lacked the third dose of pentavalent vaccine compared to 4.2 % among children aged 36–59 months (data not shown). This difference could be due to MR campaign. However, some missed opportunities from the post-campaign survey remained to be taped. As Bangladesh has high vaccination coverage, this represents a relatively large fraction of, and absolute number of, unvaccinated children.

## Discussion

Mass vaccination campaigns are considered an important strategy to increase vaccine coverage. There is on-going debate, however, regarding the potential for both positive and negative consequences of mass vaccination programs, particularly because of the targeted and time-limited nature of elimination goals and resource constraints. Thus, the aim of the present study was to assess the impact of the measles rubella campaign activities on improving population-level vaccine coverage and identify sub-group with lower coverage.

Bangladesh successfully implemented the MR campaign. In post-campaign survey, of the 4510 households interviewed, 90 % children received MR vaccine during the campaign. This coverage is high regarding the number of target population, and consistent with a study conducted in Albenia [13]. Among different divisions, Rajshahi and Rangpur had the highest (94 %) and Sylhet had the lowest (83 %) coverage which were similar to the results of the EPI coverage evaluation survey [14]. However, coverage of MCV was also increased after implementation of the campaign.

Our study result showed that age, registration prior to campaign and current school attendance of children were significantly associated with MR vaccine coverage. The strategy of inclusion of educational institutes as campaign centre for giving vaccine to the school children showed significant positive impact on MR vaccine coverage. The finding suggests that school-based campaign and delivery was more successful than the EPI center based campaign and delivery. As Bangladesh prepares for other potential school-based vaccine delivery programs, such as HPV, lessons from the MR campaign could be applied to achieve similarly or higher coverage.

The main reasons for non-vaccination during the campaign were sickness of the child, followed by fear of side effects, which are comparable the finding of another study [15]. In our study the major benefits of MR campaign according to respondents were opportunity to receive the vaccine for the children who were not previously vaccinated, massive publicity to motivate people to vaccinate their child, opportunity to acquire knowledge about measles and rubella, reduction of the fear of vaccination. A study in Uganda showed similar benefits like provision of second chance to be vaccinated and positive effect on routine immunization [16].

Our qualitative data showed that the nationwide MR campaign created positive impact to the society and the routine immunization system in spite of some limitations. The campaign increased public awareness of the intended effects of the vaccine and acceptance during both the campaign and routine EPI sessions. This finding is consistent with supplementary immunization activity (SIA) and other campaign to strengthen routine immunization [12, 17]. The campaign has strengthened the vaccine storage as well as service delivery system. These findings are also consistent with previous measles campaigns in Bangladesh [17]. The workers involved in the campaign worked beyond their designated hours to continue their routine EPI activities. So routine EPI activities were not hampered or stopped due to MR campaign which is contrast to experiences in other countries [18]. But the past experience of campaigns in Bangladesh showed similar findings as well as some other countries [17].

The main limitation of the study was that the MR vaccination information during campaign was collected through interviewing or history taking. There was no arrangement of providing card to the children/caregivers during the campaign. Even MR vaccine does not create any mark on the skin. So we had to completely rely on the recall of the respondents about the vaccination status of the children. So we used two-step confirmatory question to ensure about the vaccination status. Another limitation was the timing of the survey. We conducted post-campaign survey about 4 months later to the campaign. This delay was due to antibody detection component [unpublished observations] of this survey. But delay in conducting the survey might have created some recall bias. The length of the recall period ranged from 4 to 7 months as survey completed within 3 months from initiation.

Based on the lower coverage observed in traditional lower performing divisions, such as Sylhet, we recommend that the government and partners consider diverting additional resources to these areas in future campaigns. This could be in the form of, for example, increased supervision and monitoring or expanded demand activities.

This is the first mass campaign of a rubella containing vaccine and the MR vaccine was only recently introduced into the routine EPI system. Enhancing population awareness about rubella disease and its prevention is an important mechanism for increasing understanding of the rationale of the MR vaccine over traditional measles vaccine. We recommend that social mobilization efforts as part of both future campaigns and routine EPI focus on developing a better understanding of rubella.

Overall, the MR campaign was able to reach high levels of coverage, however, important differences existed between different subgroups of the population defined by geography. We also identified relatively low levels of interpersonal communication regarding the campaign through the registration procedure. At the same time registration was associated with a higher probability of being vaccinated. We therefore recommend that interpersonal communication through the registration procedure be a particular area of focus of future campaigns, as an approach to reducing inequalities.

While there were a range of positive impacts of the campaign on the routine EPI system, one area that we identified as a missed opportunity was the design of the campaign as a mechanism for catching up children on other incomplete vaccines. We note, however, that while the campaign was not designed as an opportunity for catch-up, some health workers did use the campaign as an opportunity to identify children who had incomplete vaccination schedules. We recommend that future campaigns be design as the opportunity to catch up on other vaccines.

## Conclusions

The results of our evaluation indicated that the campaign was successful in terms of MR coverage and in improving routine immunization services. The evaluation provided an important guideline for future evaluation efforts in Bangladesh and elsewhere.

## Abbreviations

AFRO, American Regional Office; CI, confidence interval; CES, coverage evaluation survey; EPI, expanded programme on immunization; EURO, European Regional Office; GoB, Government of Bangladesh; KII, key informant interview; MCV, measles containing vaccine; MOHFW, Ministry of Health and Family Welfare; MR, measles-rubella; OR, odd ratio; PAHO, Pan American Health Organization; SEARO-South East Asian Regional Office; SES, socioeconomic status; SIA, supplementary immunization activity; WAPRO, Western Pacific Regional Office; WHO, World Health Organization
